# Novel Complex of PD-L1 Aptamer and Albumin Enhances Antitumor Efficacy In Vivo

**DOI:** 10.3390/molecules27051482

**Published:** 2022-02-22

**Authors:** Yacong An, Xundou Li, Fengjiao Yao, Jinhong Duan, Xian-Da Yang

**Affiliations:** Institute of Basic Medical Sciences, Chinese Academy of Medical Sciences & Peking Union Medical College, Beijing 100005, China; anyacong@ibms.pumc.edu.cn (Y.A.); lixd1012@163.com (X.L.); fjyao_1103@126.com (F.Y.); jinhong_duan@aliyun.com (J.D.)

**Keywords:** PD-L1, aptamer, albumin, BSA-Apt, cancer, immunotherapy

## Abstract

The PD-1/PD-L1 pathway blockade can generate a good clinical response by reducing immunosuppression and provoking durable antitumor immunity. In addition to antibodies, aptamers can also block the interaction between PD-1 and PD-L1. For the in vivo application, however, free aptamers are usually too small in size and quickly removed from blood via glomerular filtration. To avoid renal clearance of aptamer, we conjugated the PD-L1 aptamer to albumin to form a larger complex (BSA-Apt) and evaluated whether BSA-Apt would enhance the in vivo antitumor efficacy. The PD-L1 aptamer was thiol-modified and conjugated to the amino group of BSA via a SMCC linker. The average size of BSA-Apt was 11.65 nm, which was above the threshold for renal clearance. Functionally, BSA-Apt retained the capability of the PD-L1 aptamer to bind with PDL1-expressing tumor cells. Moreover, both the free aptamer and BSA-Apt augmented the PBMC-induced antitumor cytotoxicity in vitro. Furthermore, BSA-Apt generated a significantly stronger antitumor efficacy than the free PD-L1 aptamer in vivo without raising systemic toxicity. The results indicate that conjugating the PD-L1 aptamer to albumin may serve as a promising strategy to improve the in vivo functionality of the aptamer and that BSA-Apt may have application potential in cancer immunotherapy.

## 1. Introduction

The PD1/PD-L1 blockade is one of the most promising strategies to enhance antitumor immunity. In recent years, cancer immunotherapy via the PD1/PD-L1 blockade has experienced remarkable therapeutic advances and initiated a new era of tumor treatment [1]. Immunotherapy attacks tumors by mobilizing the host’s immune system [2]. PD-1 is a coinhibitory molecule of immune response highly expressed on tumor-specific T cells [3]. PD-L1, the ligand of PD-1, is often expressed by many types of tumor cells or cells in the tumor microenvironment [4]. The engagement of PD-L1 to PD-1 suppresses T cell function, induces T cell exhaustion, and promotes tumor immune evasion [5]. The PD-1/PD-L1 blockade works by releasing the inhibitory brakes of T cells, resulting in robust activation of the immune system and durable antitumor immune responses. It has achieved dramatic clinical efficacy, especially for patients with advanced malignancies [6] and is now the first-line therapy for multiple malignancies [6]. Clinical studies have shown that the PD-1/PD-L1 blockade significantly enhances antitumor effects in various tumors, including melanoma, non-small cell lung cancer (NSCLC), renal cell carcinoma, urothelial carcinoma, head and neck squamous cell carcinoma, hepatocellular carcinoma, and microsatellite instability-high colorectal cancer [7,8,9,10,11,12]. Compared to chemotherapy, the PD-1 antibody was associated with significantly longer progression-free and overall survival in NSCLC patients with fewer adverse events [13]. Moreover, the PD-1 antibody provides durable antitumor activity in metastatic melanoma, and about 15% of patients displayed complete responses [14]. Given the unprecedented therapeutic efficacy of PD-1/PD-L1 inhibitors, the PD-1/PD-L1 axis has become a prominent target of cancer immunotherapeutics. Currently, the PD-1/PD-L1 blockade is implemented by monoclonal antibodies (mAbs). There are approximately ten approved PD-1/PD-L1 mAbs in the global market, six of which are approved by the U.S. Food and Drug Administration (FDA) [15]. However, humanized mAbs are time-consuming to produce and burdensome to store and transport [16], leading to relatively high costs of the therapeutics and limiting their availability to patients with lower incomes. Moreover, studies have shown that repeated administration of mAbs sometimes induces the production of anti-drug antibodies, reducing the mAbs’ antitumor efficacy [17]. Therefore, it is necessary to explore other types of ligands that can also block the PD-1/PD-L1 interaction.

Aptamers may potentially be utilized for the PD-1/PD-L1 blockade. Aptamers are short single-strand DNA or RNA with unique 3D structures that enable them to specifically bind with target molecules [18]. Compared with antibodies, aptamers have certain advantages, including low immunogenicity, low production cost, easy chemical modification, and better tumor penetration [19]. Previous studies have shown that aptamers can be employed as ligands in therapeutic and diagnostic applications [20,21]. Furthermore, therapeutic aptamers can function as either agonists or antagonists [22,23,24]. Macugen^®^, the first aptamer-based drug, was approved by the FDA for treatment of age-related macular degeneration in 2004 [25]. Other aptamer-based drugs, including ARC1905 (Zimura), E-10030 (Fovista), and NOX-A12, are being investigated in clinical trials [26,27,28]. These facts indicate that aptamers have application potential as specific ligands in drug development. Previous studies have shown that aptamers can be utilized as inhibitors targeting the PD-1/PD-L1 axis [22,23]. Lai et al. developed a PD-L1 antagonizing aptamer with high specificity and affinity. The aptamer could bind to both human and murine PD-L1 proteins with Kd of 4.7 nM and 72 nM, respectively. Moreover, the PD-L1 aptamer could also bind with PDL1-positive CT26 colon cancer cells and significantly suppress tumor growth in vivo without obvious toxicity [22]. These results suggest that the PD-L1 aptamer has potential in the development of tumor immunotherapeutics for clinical applications.

A major obstacle for in vivo aptamer application is that aptamers are rapidly removed from circulation via renal clearance. Most aptamers are extremely small (5nm or smaller) and prone to glomerular filtration, which tends to remove particles below 10nm [29]. As a result, the plasma concentration of free aptamers usually declines quickly after the cessation of infusion. One strategy to overcome this problem and extend the circulation time is conjugating aptamers to macromolecules, including polyethylene glycol (PEG), dendrimers, or other structures, such as lipid moieties [30,31,32,33,34]. To improve the potential for clinical application, the ideal excipient for conjugating with aptamers should have good biocompatibility and can be approved by the FDA for human use. Albumin is the most abundant protein in blood, constituting approximately 50% of the total plasma protein content [35]. Albumin has a half-life of two to three weeks before being catabolized [35]. Due to the nature of albumin, an aptamer–albumin complex may have certain advantages, including excellent biocompatibility, low cytotoxicity, low immunogenicity, and good biodegradability. Since albumin molecules usually do not leak into the glomerular filtrate [36], aptamers conjugated to albumin are protected from renal clearance and have a longer circulating time. Moreover, the size of the aptamer–albumin complex is probably appropriate for enrichment in tumor tissue via the enhanced permeability and retention (EPR) effect because the tumor neovasculature differs from that of normal tissues in microscopic anatomical architecture, resulting in extensive leakage of proper-sized nanoparticles into tumor tissue [37]. Taken together, albumin may potentially serve as an ideal excipient for conjugating with aptamers.

To date, however, albumin has not been conjugated to the PD-L1 aptamer for improving the aptamer’s in vivo function. To evaluate whether this approach could boost the efficacy of the PD-L1 aptamer, a novel albumin–aptamer complex (BSA-Apt) was constructed in this study by conjugating the thiol-modified PD-L1 aptamer to the amino group of bovine serum albumin (BSA) via the sulfo-SMCC linker. We found that compared with the free PD-L1 aptamer, BSA-Apt significantly enhanced the antitumor efficacy in vivo.

## 2. Results

### 2.1. Preparation of BSA-Apt

**Figure 1:**
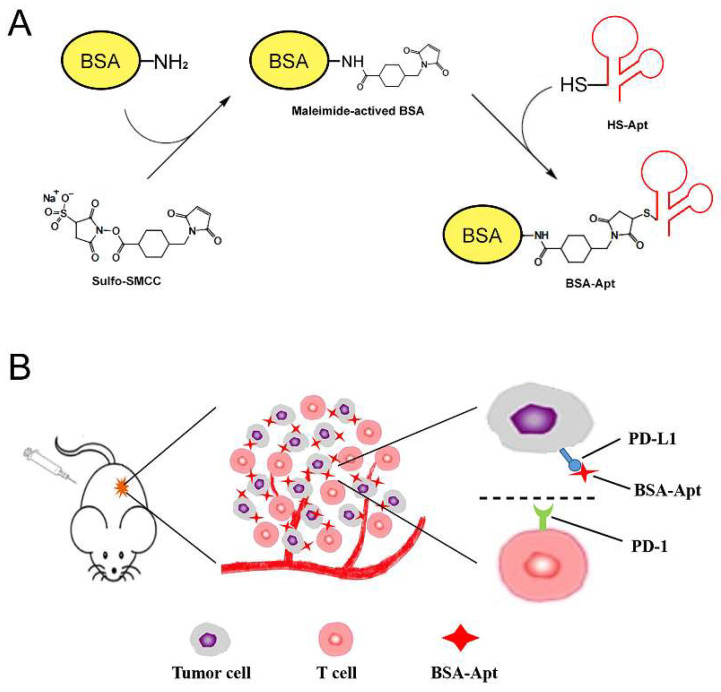
Schematic illustration of the BSA-Apt designed for colon cancer therapy. (**A**) Two-step reaction scheme for conjugating BSA with PD-L1 aptamer by a crosslinker (sulfo-SMCC). In this experiment, sulfo-SMCC first reacts with BSA to produce maleimide-activated protein. After removing non-reacted crosslinker, the maleimide-activated BSA reacts with the thiol-modified PD-L1 aptamers to form BSA-Apt. (**B**) PD-1/PD-L1 blockade scheme: BSA-Apt binds to PD-L1 expressed on the surface of tumor cells and blocks the PD-1/PD-L1 interaction.

**Figure 2:**
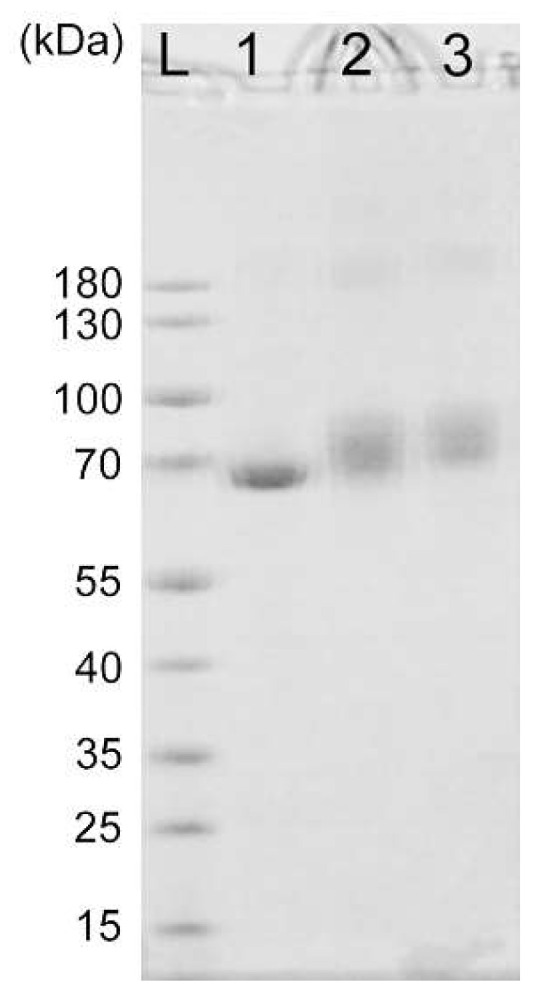
SDS-polyacrylamide gel electrophoresis of BSA. Lane L is the protein ladder. Lanes 1–3 represent images of BSA, SMCC-treated BSA, and BSA-Apt, respectively.

Previous studies have demonstrated that a PD-L1 aptamer can suppress tumor growth in vivo [22]. To overcome the challenge of renal clearance of aptamers and increase the circulating time, BSA was conjugated to the PD-L1 aptamer to form a novel nanostructure of BSA-Apt. The overall design and fabrication of BSA-Apt is illustrated in Figure 1. The thiol-modified PD-L1 aptamer was linked to the amino group of BSA via the linker sulfo-SMCC. To evaluate whether the PD-L1 aptamer was conjugated to BSA, sodium dodecyl sulfate (SDS) polyacrylamide gel electrophoresis was performed. As shown in Figure 2, the BSA-Apt band had the largest molecular weight compared with the BSA-SMCC or the BSA bands, indicating that the PD-L1 aptamer was conjugated to BSA as expected.

### 2.2. Characterization of BSA-Apt

To evaluate the aptamer conjugation efficiency (CE) and aptamer-loading rate (AL), various concentrations of thiol-modified PD-L1 aptamers were mixed with a fixed concentration of BSA-SMCC at aptamer-albumin molar ratios of 1:1, 2.5:1, and 5:1, respectively. As shown in Table 1, there were on average 0.38, 1, and 3 aptamers conjugated to each albumin molecule, respectively, with aptamer-albumin ratios of 1:1, 2.5:1, and 5:1. To facilitate multivalent binding, the subsequent experiments were conducted using the aptamer-albumin ratio of 5:1, except when specified otherwise.

**Figure 3:**
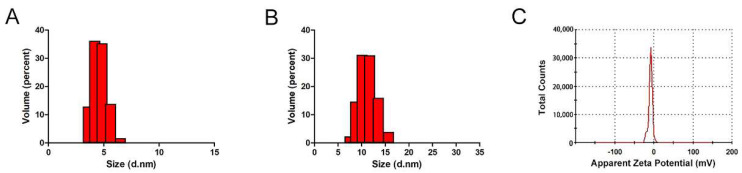
Characterization of BSA-Apt. Size distributions of free PD—L1 aptamers (**A**) and BSA—Apt (**B**). Zeta potential distribution of BSA—Apt (**C**).

The size of a nanoparticle is of critical importance for its therapeutic efficacy because particles smaller than 10nm are vulnerable to be excreted from the kidneys [38]. To evaluate the size of the BSA-Apt nanostructure, a dynamic light-scattering (DLS) study was performed. As shown in Figure 3, the BSA-Apt had an average size of 11.65 nm, while the free PD-L1 aptamer had an average size of 4.69 nm. The results indicated that the size of BSA-Apt was larger than that of the free PD-L1 aptamer. Importantly, the size of BSA-Apt is above the renal clearance threshold (approximately 10 nm), whereas the size of the free PD-L1 aptamer is below the threshold. Of note, although the size of BSA-Apt is above the renal clearance threshold, it is still relatively small to be captured by the reticuloendothelial system (RES), which tends to catch particles larger than 200 nm [39]. Moreover, the average zeta-potential of BSA-Apt was −8.32 mV (Figure 3C). The negative charges of the BSA-Apt might prevent the nanoparticles from agglomeration and maintain the stability [40].

### 2.3. Affinity of BSA-Apt to Target Cancer Cells

**Figure 4:**
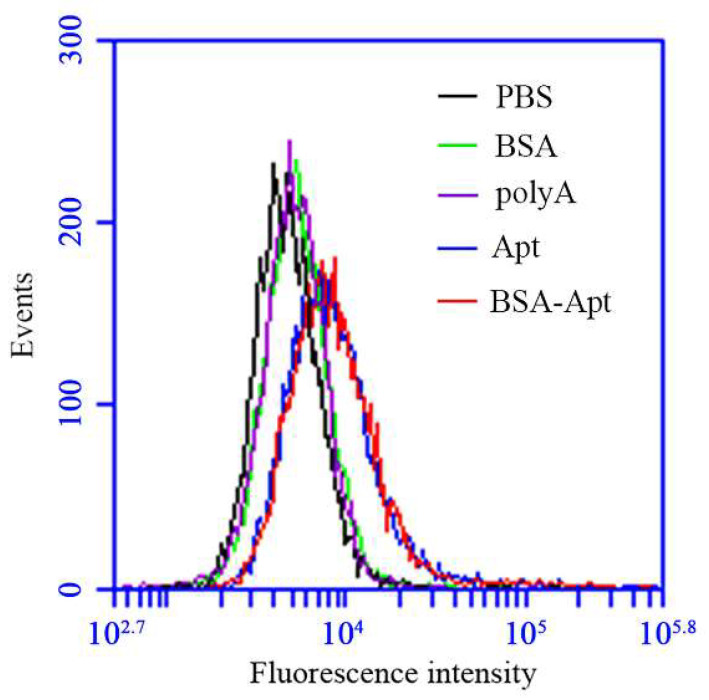
The bindings of BSA, polyA, free PD-L1 aptamer, or BSA-Apt to PDL1-positive CT26 cells. CT26 cells (2 × 10^5^) were incubated with 60 pmol of FAM-labeled BSA (green), polyA (purple), free PD-L1 aptamer (blue), or BSA-Apt (red), respectively. The cells were washed with PBS and analyzed by flow cytometry.

The PD-L1 aptamer used in this study can bind with both human and murine PD-L1 proteins [22]. As a result, the aptamer can also bind with CT26, a murine colon cancer cell line that overexpresses PD-L1 in the cell membrane [22,41]. In this study, we planned to use CT26 for the tumor model in the animal experiments and, therefore, needed to evaluate whether BSA-Apt could bind with CT26. For this purpose, FAM-labeled free PD-L1 aptamers, BSA-Apt, BSA, or polyA DNA were incubated with CT26 cells, which were evaluated by flow cytometry. To ensure a fair comparison among monovalent bindings, the BSA-Apt used here was constructed using an aptamer-albumin ratio of 2.5:1, so that each albumin was conjugated to one aptamer on average (Table 1). As presented in Figure 4, both the free aptamer and BSA-Apt generated strong fluorescent signals in CT26 cells, while BSA and polyA generated a very weak signal. The results indicated that the aptamer could still bind with CT26 cells after being conjugated to BSA.

**Figure 5:**
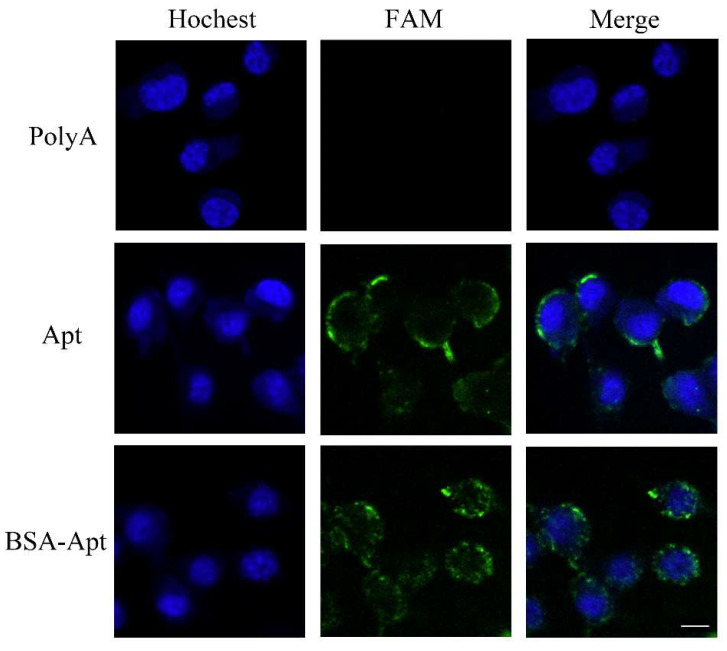
Confocal microscopy evaluation of CT26 cells stained by free PD-L1 aptamer or BSA-Apt. Green fluorescence signals were generated by FAM-labeled polyA, free PD-L1 aptamer, or BSA-Apt. The nuclei were stained blue with Hoechst. Bar represents 10 μm.

To further investigate whether BSA-Apt could bind to CT26 colon cancer cells, confocal microscopy was used to study the CT26 cells treated by FAM-labeled PD-L1 aptamer, BSA-Apt, or polyA that served as a control DNA sequence. As shown in Figure 5, both BSA-Apt and the free PD-L1 aptamer generated strong fluorescent signals, while polyA produced barely any fluorescence. The results again indicated that BSA-Apt could bind to CT26 cells.

### 2.4. BSA-Apt Enhanced PBMC’s Cytotoxicity against Tumor Cells In Vitro

**Figure 6:**
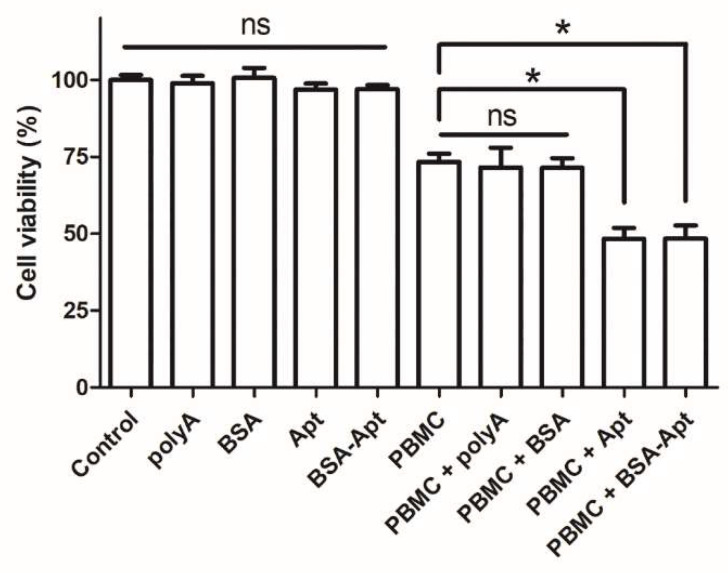
Immune cytotoxicity against PDL1-positive MDA-MB-231 cells in vitro. MDA-MB-231 cells were cultured in the presence or the absence of PBMC. The cells were treated with polyA, BSA, PD-L1 aptamer, or BSA-Apt. The PBMC was subsequently washed off and tumor cells were evaluated for viability with standard MTS assay (*n* = 6). Single star indicates statistically significant differences (*p* < 0.05), and “ns” indicates no significance.

Multiple studies have established that MDA-MB-231 cells overexpress PD-L1 in the cell membrane [42,43]. Moreover, the efficacy of the immune checkpoint blockade can be estimated in vitro by PBMC-induced cytotoxicity against MDA-MB-231 cells, with/without the PD-1/PD-L1 inhibitors in the medium [44,45,46]. The PD-L1 aptamer has been shown to enhance the antitumor immunity via blocking the PD-1/PD-L1 axis [22]. Here in this study, because the PD-L1 aptamer was conjugated to BSA via the SMCC linker, it was unknown whether BSA-Apt could retain the same blocking function. To address this issue, PDL1-positive MDA-MB-231 cells were co-cultured with PBMC and treated with BSA, the free PD-L1 aptamer, BSA-Apt, or polyA that served as a control DNA in this experiment. As shown in Figure 6, in the absence of PBMC, polyA, BSA, the free PD-L1 aptamer, or BSA-Apt did not influence the viability of the tumor cells. However, in the presence of PBMC, both PD-L1 aptamers and BSA-Apt significantly enhanced PBMC-mediated cytotoxicity against the tumor cells, whereas BSA *per se* or polyA failed to induce any potentiation. The results suggested that, similar to the PD-L1 aptamer, BSA-Apt could also enhance the PBMC-induced cytotoxicity to tumor cells in vitro, presumably by antagonizing the PD-1/PD-L1 mediated immunosuppressive effects.

### 2.5. BSA-Apt Generated Superior Antitumor Efficacy In Vivo

**Figure 7:**
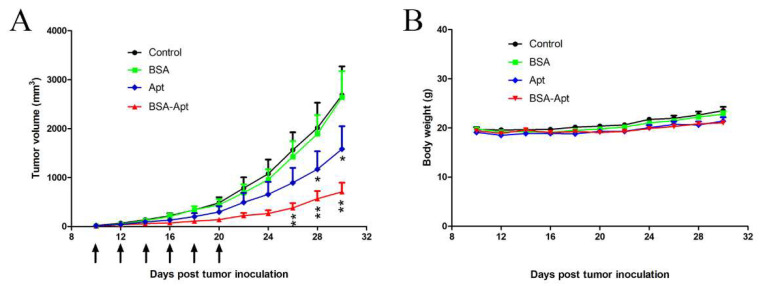
In vivo tumor inhibition by various treatments. CT26-bearing BALB/c mice were divided into four groups, with eight animals in each group. The mice were treated with PBS (control), BSA (1.6 mg/kg), free PD-L1 aptamers (1.2 mg/kg), or BSA-Apt (with BSA of 1.6 mg/kg and aptamer of 1.2 mg/kg). The treatments were administrated by intraperitoneal injections every two days, for a total of six injections (arrows). (**A**) Tumor volume and (**B**) body weight were recorded and shown. Single star indicates statistically significant differences (*p* < 0.05) between the free aptamer and the control groups, and double stars indicate significant differences (*p* < 0.05) between the free aptamer and the BSA-Apt groups.

Although BSA-Apt could enhance PBMC-induced cytotoxicity to target cells in vitro, it was unclear whether BSA-Apt could generate antitumor efficacy in vivo. To address this issue, mice bearing CT26 tumors were treated with PBS, BSA, the free PD-L1 aptamer, or BSA-Apt via intraperitoneal injections every two days, for a total of six injections. As shown in Figure 7A, BSA treatment had no obvious antitumor effects, and tumor growth was not suppressed. However, the free PD-L1 aptamer inhibited tumor growth to a certain degree. Importantly, BSA-Apt generated the most marked antitumor efficacy, which was statistically different from that generated by the free PD-L1 aptamer. Moreover, body weight measurements did not show significant differences among the groups (Figure 7B), indicating that all treatment groups had similar toxicity profiles. The results suggested that BSA-Apt enhanced the antitumor efficacy of the PD-L1 aptamers in vivo without generating extra systemic toxicity.

## 3. Discussion

In this study, a new nanostructure (BSA-Apt) was evaluated for the first time to improve the antitumor efficacy of a PD-L1 aptamer. BSA-Apt was constructed by conjugating the thiol-modified PD-L1 aptamer to the amino group of BSA via the linker sulfo-SMCC (Figure 1 and Figure 2). The average diameter of the BSA-Apt was about 11.65 nm (Figure 3). Similar to the free PD-L1 aptamer, BSA-Apt could bind to the PDL1-positive tumor cells (Figure 4 and Figure 5). Moreover, BSA-Apt enhanced PBMC-induced cytotoxicity against tumor cells in vitro (*p* < 0.05), presumably by mitigating PD-1/PD-L1-mediated immunosuppression (Figure 6). Furthermore, compared with free PD-L1 aptamers, BSA-Apt generated stronger antitumor efficacy in vivo (*p* < 0.05) without raising the systemic toxicity (Figure 7). These results suggested that BSA-Apt may have better application potential in immunotherapy against colon cancer.

Free aptamers usually have a short lifetime in blood owing to rapid renal clearance, creating a major obstacle for in vivo aptamer application. An aptamer of 15–50 nt (5–15 kDa MW) has a size smaller than 5 nm and thus, can be excreted by kidneys capable of removing substances with a molecular weight below 30–50 kDa [47,48]. To address this issue, aptamers are often formulated by conjugating with bulky moieties, including polyethylene glycol (PEG) [49], dendrimers [50], avadin [51], or cholesterol [52], to reach a mass above the threshold of glomerular filtration. PEGylation is a useful method to overcome renal clearance of aptamers. However, a number of reports have documented the development of anti-PEG antibodies following treatment with PEGylated therapeutics, which are associated with reduced efficacy and hypersensitivity [53,54,55]. In addition, due to the non-biodegradability of PEG, there may be unforeseen biological effects, such as PEG-related vacuolation [56]. Dendrimers with cationic amino surface groups, such as poly(amido amine) (PAMAM) or poly(propylene imine) (PPI), are also employed to bind with aptamers to prevent renal clearance [50]. However, cationic dendrimers have a typical dose-dependent cytotoxicity that cannot be ignored. The interaction between positively charged dendrimers with negatively charged biological membranes tends to destabilize the cell membrane through nanopore development, leading to leakage of cellular content and subsequent cell lysis [57,58]. Therefore, it is necessary to explore other types of excipients with good biocompatibility and biodegradability for conjugating with aptamers. Albumin is a water-soluble protein with a molecular weight of 69 kDa and does not leak much into the glomerular filtrate. It is the most abundant protein in plasma and has a half-life of two to three weeks before being catabolized by the reticuloendothelial system [35]. As a result, albumin is considered a molecule with excellent biocompatibility and biodegradability. Moreover, recombinant human albumin has been approved by the FDA for clinical use. In this study, BSA-Apt exhibited a stronger antitumor efficacy than the PD-L1 aptamer *per se* (Figure 7). The results indicated that conjugating albumin with the PD-L1 aptamer may serve as a strategy to enhance aptamer functionality for in vivo applications. Of note, BSA may potentially induce an immune response in mice with long-term therapy. If conjugating PD-L1 aptamer to albumin is further developed for clinical application, recombinant human albumin should be used to minimize the potential immunogenicity.

The improvement of in vivo anticancer efficacy by BSA-Apt may be related to several factors (Figure 7). First, based on the biokinetics, a nanoparticle with size of 10–150 nm is optimal for in vivo application because it can escape renal clearance and avoid uptake by the reticuloendothelial system (RES) of the spleen and liver, which tend to capture particles larger than 200 nm [59]. Here in this study, BSA-Apt has an average size of 11.65 nm (Figure 3), which is suitable to prolong the circulating time. Second, the size of BSA-Apt is also appropriate for drug enrichment in tumor tissue via the EPR effect, which is an unique phenomenon of solid tumors with high vascular density because large gaps exist between endothelial cells in tumor blood vessels, leading to selective extravasation and retention of macromolecular drugs or nanoparticles in tumor tissue [60]. Third, each BSA-Apt may have multiple aptamers with the potential to generate multivalent binding to target tumor cells, further improving the tumor-targeting effect. Taken together, conjugating the PD-L1 aptamers to albumin may prolong circulating time and improve tumor-targeting via the ERP effect, resulting in augmented anticancer efficacy in vivo.

To improve the potential for clinical application, it is preferable that all the components of a drug candidate are prospectively approvable by the regulatory agencies. The albumin–aptamer conjugate has the potential of immunotherapeutics for the following reasons: First, albumin is the most abundant protein in plasma with excellent biocompatibility. Albumin is a major component of Abraxane^®^, which has been approved by the FDA to treat breast cancer [40]. Second, aptamer is a single-strand nucleic acid and usually has good biodegradability. An aptamer drug (Macugen^®^) has been approved by the FDA for clinical usage in 2004 [61]. Third, the SMCC linker, utilized here for conjugating BSA with the PD-L1 aptamer, has been approved by the FDA for usage in T-DM1^®^, an antibody-drug conjugate (ADC) for HER2-targeting chemotherapy [62]. Moreover, the process of BSA-Apt conjugation was relatively uncomplicated and potentially scalable. Taken together, BSA-Apt is a nanostructure with good biocompatibility and biodegradability, low cytotoxicity, as well as production scalability.

In summary, the PD-1/PD-L1 blockade has proven to be an effective modality to overcome immunosuppression and provoke robust and durable antitumor responses [63]. In this study, a novel nanostructure (BSA-Apt) was constructed by conjugating albumin to the PD-L1 aptamer. Compared with the free PD-L1 aptamer, BSA-Apt significantly improved antitumor efficacy in vivo, and may have application potential in cancer immunotherapy.

## 4. Materials and Methods

### 4.1. Cell Lines and Cell Culture

CT26 (mouse colon cancer cell line) and MDA-MB-231 (human triple-negative breast cancer cell line) were obtained from the Cell Center of Chinese Academy of Medical Sciences (Beijing, China). The peripheral blood mononuclear cells (PBMCs) were isolated from healthy donors by using a lymphocyte separation medium (TBD, Tianjin, China) following the manufacturer’s protocol [64]. CT26 cells were cultured in DMEM medium (Gibco). PBMC and MDA-MB-231 cells were cultured in RPMI-1640 medium (Gibco). The cell culture medium was supplemented with 10% fetal bovine serum (FBS), 100 U/mL penicillin, and 100 μg/mL streptomycin. Cells were grown in a humid atmosphere with 5% CO_2_ at 37 °C. All donors were required to sign an informed consent. The protocol was approved by the Ethics Committee of Chinese Academy of Medical Sciences and Peking Union Medical College, and all methods were conducted in accordance with the Declaration of Helsinki.

### 4.2. Animals

BALB/c female mice (6–8 weeks old, 18–22 g) were purchased from Beijing Vital River Laboratory Animal Technology Co., Ltd. (Beijing, China). All mice were fed with standard diets and water. The animal study and procedures were approved by the Ethics Committee of Institute of Basic Medical Sciences, Chinese Academy of Medical Sciences, according to the institutional animal care and use guidelines.

### 4.3. Reagents

The PD-L1 aptamer with a 5′ polyT linker and 5′-SH modification and the sequence of 5′-SH-TTTTTT-ACGGGCCACATCAACTCATTGATAGACAATGCGTCCACTGCCC GT-3′ were synthesized by Invitrogen (Shanghai, China). Bovine serum albumin (BSA) was purchased from TBD Science Bio-engineering Co., Ltd. (Tianjin, China). Tris (2-carboxyethyl) phosphine (TCEP) was purchased from Sigma-Aldrich (Shanghai, China). Sulfosuccinimidyl 4-[*N*-maleimidomethyl]cyclohexane-1-carboxylate (sulfo-SMCC) was purchased from Aladdin Chemical Co., Ltd. (Shanghai, China).

### 4.4. Conjugation of Aptamer to Albumin

For the preparation of BSA-Apt, sulfo-SMCC, a commonly used linker, was utilized to conjugate the thiol-modified PD-L1 aptamer to the amino groups of albumin [65]. Briefly, 1 mg of BSA and 0.9 mg of sulfo-SMCC were incubated in 1 mL PBS (100 mM, pH 7.2) for 30 min at room temperature. Excess sulfo-SMCC was removed by ultrafiltrating with a device of 30 kD cut-off, and the SMCC-treated BSA was resuspended in 200 μL PBS. Subsequently, 240 μg of thiol-modified PD-L1 aptamer powder was dissolved in 360 μL PBS, and 40 μL 800 mM of TCEP solution was added to expose the sulfhydryl group for 1 h. The SMCC-treated BSA solution was mixed with the aptamer solution, and the reaction mixture was incubated at room temperature overnight. The product (BSA-Apt) was ultrafiltrated with a device of 30KD cut-off and resuspended in PBS.

### 4.5. Assessment of DNA Conjugation to BSA

Sodium dodecyl sulfate polyacrylamide gel electrophoresis (SDS-PAGE) experiments were used to evaluate whether aptamers were conjugated to BSA. The 4–12% precast polyacrylamide gel (Invitrogen, Shanghai, China) was used to distinguish BSA and BSA-Apt. An amount of 2 μg of BSA, SMCC-treated BSA, or BSA-Apt were loaded into the gel. The samples were subjected to 180 V for 1 h. The BSA was visualized by Coomassie Blue staining.

### 4.6. Measurement of Aptamer Conjugation Efficiency and Aptamer-Loading Rate

For estimation of the percentage of PD-L1 aptamer conjugated to albumin, a fixed concentration of BSA-SMCC was mixed and reacted with thiol-modified PD-L1 aptamer at albumin-aptamer molar ratios of 1:1, 1:2.5, and 1:5, respectively. The solution was ultrafiltrated with a device of 30KD cut-off that could retain albumin. The DNA content in the ultrafiltrate was measured via NanoDrop 2000 (Thermo Fisher, Waltham, MA, USA). Pierce bicinchoninic acid (BCA) assay was utilized for BSA quantitation in BSA-Apt [66] per manufacturer’s instruction. All experiments were repeated at least three times. The aptamer conjugation efficiency (CE) and aptamer-loading rate (AL) were calculated by the following equations:

$$\mathrm{CE}\;\left(\%\right)=\frac{\mathrm{total}\;\mathrm{aptamer}\;\mathrm{added}\;-\;\mathrm{unconjugated}\;\mathrm{aptamer}}{\mathrm{total}\;\mathrm{aptamer}\;\mathrm{added}}\;\times \;100\%$$

$$\mathrm{AL}\;\left(\%\right)=\frac{\mathrm{number}\;\mathrm{of}\;\mathrm{aptamer}\;\mathrm{added}\;–\;\mathrm{number}\;\mathrm{of}\;\mathrm{unconjugated}\;\mathrm{aptamer}}{\mathrm{number}\;\mathrm{of}\;\mathrm{albumin}}\;\times \;100\%$$

### 4.7. Characterization of BSA-Apt

The average particle size and zeta potential of the BSA-Apt nanoparticles were evaluated by dynamic light scattering (DLS), using Zeta Sizer Nano ZS90 (Malvern Instruments, Malvern, UK) at 25 °C.

### 4.8. Evaluation of Cellular-Binding Capacity

CT26 cells used in the experiments were all in a logarithmic growth phase. CT26 cells (2 × 10^5^) were incubated with 60 pmol of FAM-labeled BSA, poly-A, the free PD-L1 aptamer, or BSA-Apt in 300 uL of PBS for 30 min at room temperature, then washed twice with 200 uL of PBS, resuspended in 200 uL PBS, and analyzed by flow cytometry (Accuri C6 Flow Cytometer, BD, San Jose, CA, USA).

### 4.9. Confocal Imaging Studies

CT26 cells were cultured in Lab-Tek Chamber #1.0 Borosilicate Coverglass System (ThermoFisher, Waltham, MA, USA) at a density of 6 × 10^4^ cells/mL for 12 h. The cells were incubated with FAM-labeled poly-A, the free PD-L1 aptamer, or BSA-Apt at an equivalent dose of ssDNA (100 pmol) for 4 h and washed thrice with PBS. Cells were fixed with 4% paraformaldehyde solution for 10 min at 4 °C. After washing cells twice again with PBS, cells were stained with Hoechst (Solarbio, Beijing, China) for 30 min at room temperature. Finally, cells were washed thrice with PBS, incubated with 100 uL PBS, and analyzed by a confocal laser-scanning microscope (Perkin Elmer Ultraview, Perkin, Waltham, MA, USA).

### 4.10. In Vitro Cytotoxicity Assays

MDA-MB-231 tumor cells (1 × 10^4^) were grown in 96-well plates and mixed with PBMC at an effector:target ratio (E:T) of 5:1. The mixture or the MDA-MB-231 cells were treated with BSA, polyA, the free PD-L1 aptamer, or BSA-Apt at a dose of 40 pmol ssDNA. After incubating at 37 °C for 72 h, the MDA-MB-231 cells were washed thrice with PBS. Cell viability was determined by MTS assay according to the standard protocol as outlined by the manufacturer (Promega, Madison, WI, USA).

### 4.11. In Vivo Antitumor Study

For the murine colon cancer model, 2 × 10^5^ CT26 cells suspended in 100 μL PBS were injected subcutaneously on the right rear flank of BALB/c mice. When tumor diameter reached about 5 mm, mice were randomly divided into four groups. The mice were treated with PBS, BSA, the free PD-L1 aptamer, or BSA-Apt via intraperitoneal injection every two days for a total of six injections. The dosages of the PD-L1 aptamer and BSA-Apt were 1.2 mg/kg of ssDNA per animal. Animals in the BSA group received the same amount of albumin (1.6 mg/kg) as the BSA-Apt group. Tumor size and body weight were measured every two days during the experiment. Tumor volume was calculated according to the formula (a × b^2^)/2, where a and b represent the length and width of the tumor, respectively.

### 4.12. Statistical Analysis

Statistical analyses were performed by GraphPad Prism 5 software (La Jolla, CA, USA). One-way ANOVA with Fisher’s least significant difference (LSD) comparison test was used for statistical calculation. *p* < 0.05 was regarded as statistically significant. All data are presented as mean value with standard deviation indicated (mean ± SD).

## Figures and Tables

**Table 1:** CE and AL for BSA-Apt.

Aptamer-albumin molar ratio	1:1	2.5:1	5:1
Aptamer conjugation efficiency (CE)	37.11 ± 3%	43.19 ± 1.46%	58.49 ± 1.23%
Aptamer-loading rate (AL)	0.37 ± 0.03	1.08 ± 0.04	2.92 ± 0.06

Note: Each value presents mean ± SD (*n* = 3).

## Data Availability

The data presented in this study are available in this article.

## Sample Availability

Samples of the compounds are not available from the authors.
